# Synergistic Protection of N-Acetylcysteine and Ascorbic Acid 2-Phosphate on Human Mesenchymal Stem cells Against Mitoptosis, Necroptosis and Apoptosis

**DOI:** 10.1038/srep09819

**Published:** 2015-04-24

**Authors:** Chia-Jung Li, Li-Yi Sun, Cheng-Yoong Pang

**Affiliations:** 1 Institute of Medical Sciences, Tzu Chi University, Hualien, Taiwan; 2 Department of Medical Research, Buddhist Tzu Chi General Hospital, Hualien, Taiwan

## Abstract

Human mesenchymal stem cells (hMSCs) contribute to ischemic tissue repair, regeneration, and possess ability to self-renew. However, poor viability of transplanted hMSCs within ischemic tissues has limited its therapeutic efficiency. Therefore, it is urgent to explore new method to improve the viability of the grafted cells. By using a systematic analysis, we reveal the mechanism of synergistic protection of N-acetylcysteine (NAC) and ascorbic acid 2-phosphate (AAP) on hMSCs that were under H_2_O_2_-induced oxidative stress. The combined treatment of NAC and AAP (NAC/AAP) reduces reactive oxygen species (ROS) generation, stabilizes mitochondrial membrane potential and decreases mitochondrial fission/fragmentation due to oxidative stress. Mitochondrial fission/fragmentation is a major prologue of mitoptosis. NAC/AAP prevents apoptotic cell death via decreasing the activation of BAX, increasing the expression of BCL2, and reducing cytochrome *c* release from mitochondria that might lead to the activation of caspase cascade. Stabilization of mitochondria also prevents the release of AIF, and its nuclear translocation which may activate necroptosis via H2AX pathway. The decreasing of mitoptosis is further studied by MicroP image analysis, and is associated with decreased activation of Drp1. In conclusion, NAC/AAP protects mitochondria from H_2_O_2_-induced oxidative stress and rescues hMSCs from mitoptosis, necroptosis and apoptosis.

Human mesenchymal stem cells (hMSCs) are multipotent stromal cells derived from mesenchymes that reside within the bone marrow and adipose tissue. Currently, human bone marrow-derived mesenchymal/stromal cells (hBMSCs) have been widely tested in treating various diseases, for instance as an immune-modulator in allogenic bone marrow transplantation^1^,^2^. However, the advantages of human adipose tissue-derived mesenchymal stem cells (hADMSCs), such as minimal patient discomfort during procurement and expand more rapidly, have drawn the attention of using them as a more ideal source of MSCs for autologous cells transplantation^3^,^4^.

MSCs from various tissues can be easily isolated, however the low survival rate and increased cell death after implantation into the ischemic/injured tissues suggest that the microenvironment may not be conducive to their viability^5^,^6^,^7^. Excessive production of reactive oxygen species (ROS) due to sustained oxidative stress in ischemia tissues is an essential factor that affects the survival of engrafted MSCs^6^,^7^. ROS are formed as a natural byproduct of the normal energy metabolism. ROS have been shown to play key role in the growth and homeostasis of MSCs: lower ROS resulted in enhancement of proliferation, survival and differentiation, while excessive ROS could lead to mitochondrial dysfunction, cell death, tissue inflammation, and the aging of hMSCs by potentially compromising their differentiation and regeneration ability^8^,^9^,^10^,^11^,^12^. Furthermore, mitochondrial dysfunction has been suggested to be the main cause of oxidative stress-induced apoptosis and necrosis during ischemia-reperfusion injury^13^,^14^. Therefore, protecting mitochondria and enhancement of cell survival is one of the important measures in the development of hMSCs-based cytotherapy for ischemic tissue injury^6^,^7^,^15^.

L-Ascorbic acid 2-phosphate (AAP) is an oxidation-resistant derivative of ascorbic acid. AAP has been shown to promote mammalian cell differentiation and DNA synthesis^16^,^17^. N-acetyl-L-cysteine (NAC) is a prodrug/precursor of biologic antioxidant, glutathione (GSH). Thus, NAC can serve as a potent ROS inhibitor, and has been widely used to counter the adverse effects arising from oxidative stress^18^. On the other hand, hypoxia has been shown to affect the secretion of several growth factors, such as VEGF, HGF, HIF, and FGF-2, which all have been shown to accelerate the proliferation of MSCs^19^,^20^.

Our previous study has also demonstrated that combined treatment of NAC and AAP (NAC/AAP) promotes cell proliferation by suppressing cyclin-dependent kinase inhibitors in hADMSCs. These NAC/AAP-treated hADMSCs retained their stem cell properties (as revealed by the upregulation of several stemness genes), and their differentiation potential^21^. Moreover, these NAC/AAP-induced changes were quite similar to MSCs cultivated under hypoxia (1%–5% pO_2_)^22^.

However, the mechanism by which NAC/AAP treatment in helping cells to counter oxidative stress is still not fully elucidated. In this study, we systematically tested 32 different combinations of NAC and AAP to find out the optimized concentration that produced maximum protection for hMSCs suffering from oxidative stress. We then clarified the major signal transduction pathways that were responsible for the survival of hMSCs that were pretreated with NAC/AAP.

## Results

### NAC/AAP protected hADMSCs against H_2_O_2_-induced cell death

**Figure 1:**
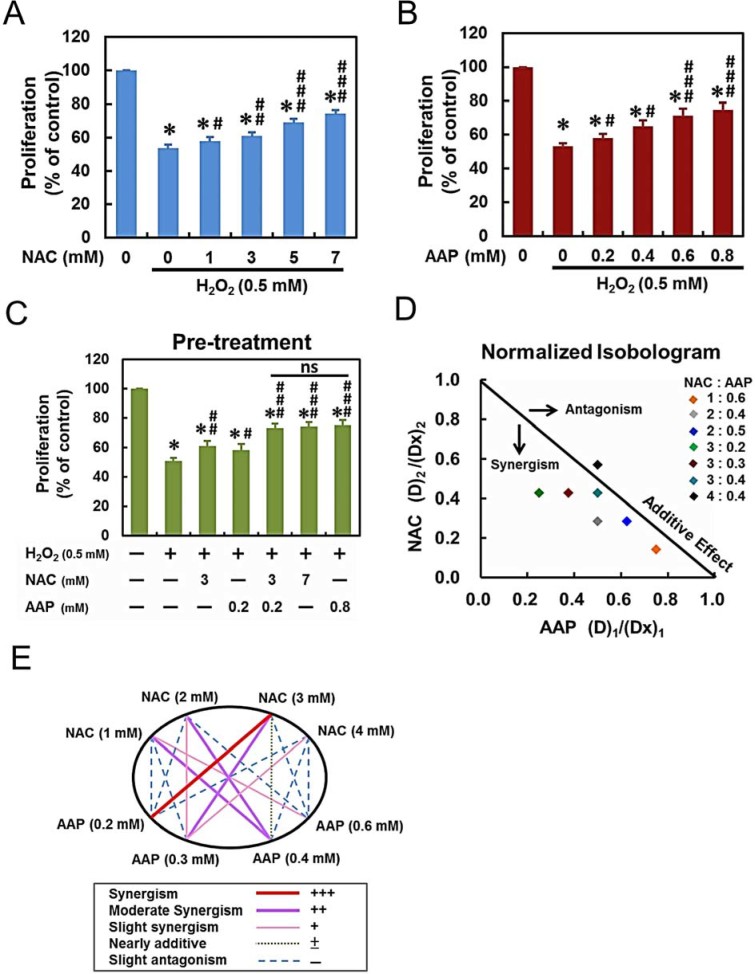
NAC/AAP inhibited H_2_O_2_-induced growth inhibition in hADMSCs. (A and B) hADMSCs were pretreated with different concentrations of NAC or AAP followed by stimulation of 0.5 mM H_2_O_2_ for 4 h, and the cell proliferation was assessed by Alamar Blue assay. (C) hADMSCs were pretreated to different concentrations of NAC and/or AAP against ROS-inhibited cell proliferation. Combined pretreatment resulted in significant growth increased of hADMSCs, more than that by either drug alone; the degree of proliferation in high-dose alone groups were not significant. (D) Post-treatment of cells with NAC and/or AAP did not affect the proliferation by H_2_O_2_ at 24 and 48 h (E) Isobologram analysis of protective effects of NAC/AAP pretreatments alone or in combination against H_2_O_2_-induced cytotoxicity in hADMSCs. The diagonal line represents the isoeffect line of additivity. A combination index of 1.0 (solid line) reflects additive effects, whereas values greater than and less than 1.0 indicate antagonism and synergy, respectively. (F) Graphical representation of combinatorial dosing. All dosing combinations show synergy as determined by the Chou-Talalay method. * *p* < 0.001, as compared to the control. # *p* < 0.05; ## *p* < 0.01; ### *p* < 0.001, as compared to the H_2_O_2_-treated group. ns, not significant.

In this study, we used H_2_O_2_ as an oxidative stressor to evaluate its effects on hADMSCs, the adipose tissue-derived MSCs. Treatment with various concentrations of H_2_O_2_ for 4 h reduced the hADMSCs proliferation in a dose-dependent manner, and the median effective dose (ED_50_) was determined to be approximately 0.5 mM. To test whether NAC and/or AAP were able to protect hADMSCSs from oxidative stress, we treated hADMSCs with various concentrations of NAC and/or AAP for 20 h followed by exposure to 0.5mM H_2_O_2_ for 4 h (Fig. 1A & B). As shown in Figure 1A, hADMSCs were significantly rescued by NAC in a dose-dependent manner (*p <* 0.001). AAP also rescued the survival of hADMSCs in a dose-dependent manner (Fig. 1B, *p <* 0.001). To evaluate the synergistic protecting effect of NAC and AAP, we subjected hADMSCs to various NAC/AAP concentrations for the subsequent studies. The proliferation of combined treatment was similar to that observed in high-dose single treatment groups (NAC: 7mM; AAP: 0.8mM, respectively). And there was no significant difference of cell proliferation between the high-dose groups and the combination of low-dose (Fig. 1C).

Analysis of the protection obtained by NAC and AAP co-treatment indicated a synergistic effect as revealed by the isobologram: most of the data points located below the line of additive effects (Fig. 1D). The analysis reflected that NAC/AAP yielded a better protection than either drug alone. Figure 1E showed a normalized isobologram for various tested values in hADMSCs. The combination index (CI) curve shown in Fig. 1E also demonstrated the synergistic protection of NAC when combined with AAP, with CI values ranging from 0.679 to 1.96 at various drug combinations. CI values that are < 1 in tested doses indicating synergy, and tested doses show an improvement over single doses. The combined treatment of 1mM NAC and 0.2mM AAP obtained the best score in DRI (Supplementary Table S1), however, the combination of 3mM NAC and 0.2mM AAP reached the best score in CI (Fig. 1E).

### NAC/AAP protected hADMSCs from H_2_O_2_-induced apoptosis and necrosis

**Figure 2:**
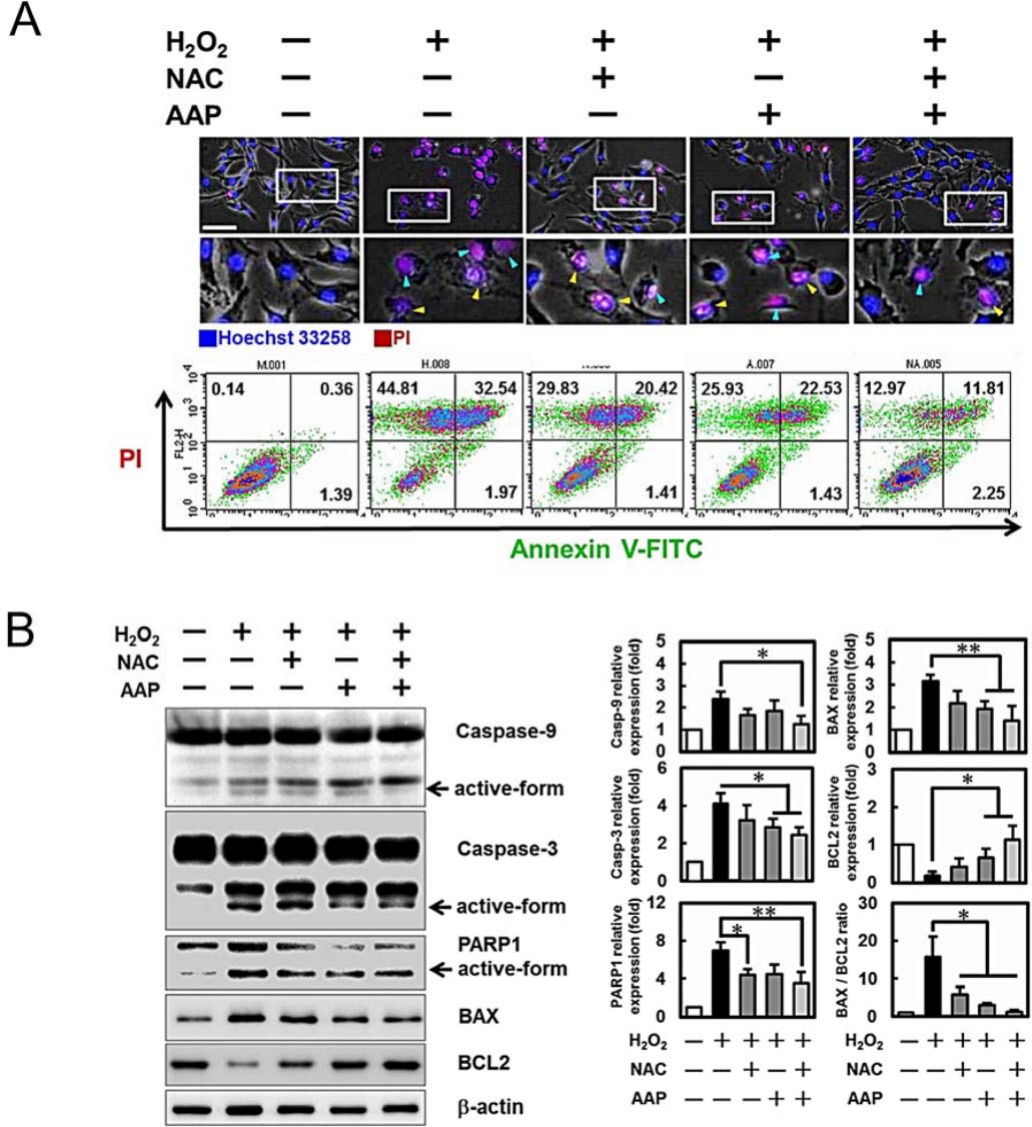
NAC/AAP protected hADMSCs against H_2_O_2_-induced apoptosis and necrosis. A, upper panel: cells were stained with Hoechst 33258 (blue) and PI (red) for live-cell imaging and monitoring. Blue arrowheads indicate necrotic cells; yellow arrowheads indicate apoptotic and necrotic-like cells. To determine whether NAC and/or AAP inhibition of H_2_O_2_-induced cell death was associated with apoptosis and necrosis (A, lower panel), Annexin-V/PI double-staining was used to detect the PS out-flipping phenomenon and analysed by flow cytometry. (B) Whole cell lysates isolated from hADMSCs of various treatments as indicated were analyzed for caspase-3 and -9, PARP, BAX, and BCL2 expressions. The protein expression levels were normalized to b-actin and expressed as the fold change to the respective control. Data are representative of 3 independent experiments. Scale bar = 100 µm. * *p* < 0.05 and ** *p* < 0.01 compared to the H_2_O_2_-treatment control.

The nuclei of hADMSCs after various treatments were observed by live-cell fluorescent microscopy after staining with Hoechst dye and PI. No abnormal cell was found in the control group while a high number of cells displayed typical apoptosis- (nuclear shrinkage, apoptotic bleb, and irregular shape) and necrosis-like changes (cell swelling, plasma membrane rupture, and detachment; minor population as compared to the apoptotic cells) were noted in H_2_O_2_ treated cells (photomicrographs in Fig. 2A). Flow cytometric analysis (Fig. 2A lower panel) also showed that PI^+^ cells were decreased in the NAC/AAP treated cells subjected to H_2_O_2_ challenge. Among them, the Annexin V^+^/PI^+^ cells were indicated as the late apoptotic cells (NAC/AAP pretreated 11.81% *vs.* non-treated 32.54%). To evaluate whether NAC/AAP attenuated the cell death of hADMSCs by reducing the ROS generation in the present study, the intracellular ROS level was examined using DCFDA and DHE staining. Our data show that NAC/AAP protected hADMSCs from oxidative stress injury, at least partially, by inhibiting the intracellular ROS generation (Fig. S1).

The activation of caspases and expression of pro-apoptotic proteins were revealed by immunoblotting (Fig. 2B). Besides increasing the expression of the anti-apoptotic BCL2, NAC/AAP decreased the expressions of BAX, cleaved caspase-9, -3, and PARP1. NAC/AAP also markedly decreased the ratio of BAX/BCL2 protein ratio. These findings indicated that activation of caspase cascade could be one of the mechanisms of H_2_O_2_‐induced apoptosis and necrosis in hADMSCs.

### NAC/AAP suppressed mitochondrial dysfunction during H_2_O_2_-induced cell death

**Figure 3:**
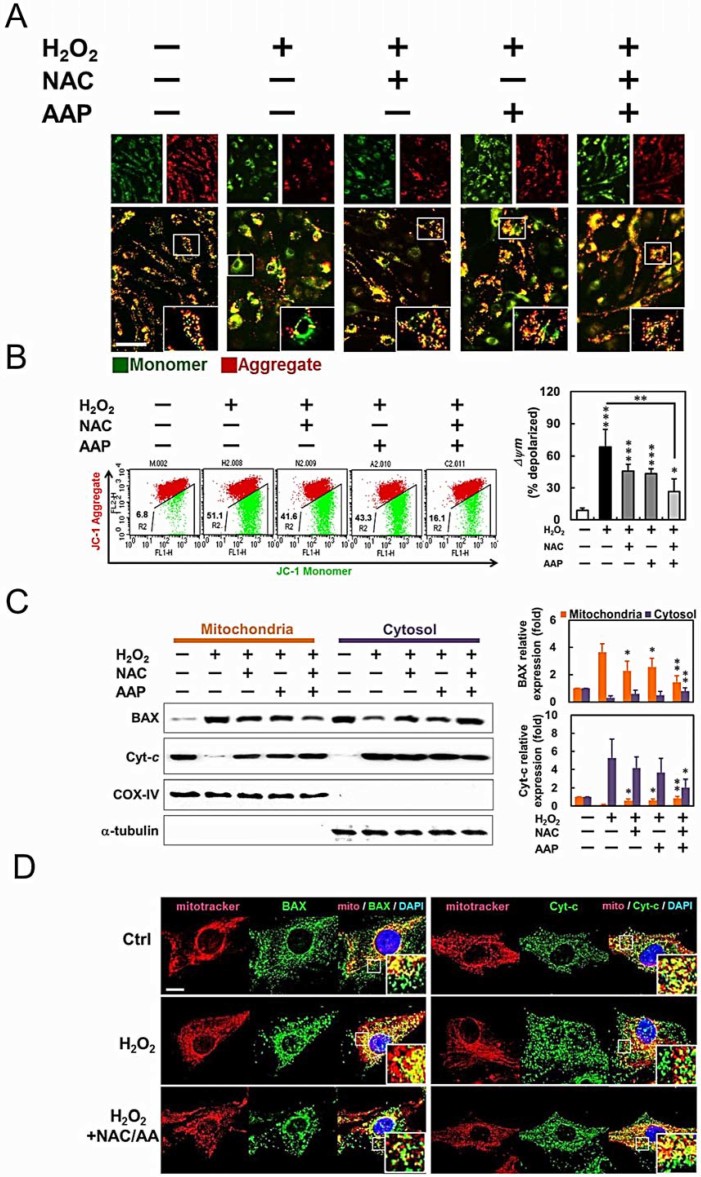
NAC/AAP protected mitochondria from H_2_O_2_-induced apoptosis. (A) The MMP was measured using JC-1 fluorescence imaging in hADMSCs. The JC-1 monomer was represented by green fluorescence, the JC-1 aggregate image was represented by red fluorescence, and the merged images were the combined of the green and red images. Control cells showed strong aggregated red fluorescence indicative of normal membrane potential. Scale bar = 100 mm. (B) Changes of MMP in NAC or/and AAP-pretreated hADMCS by flow cytometry. Fluorescence intensity shifted from the higher level to the lower one indicates the loss of MMP. Mitochondria depolarization is indicated by an increase in the red fluorescence intensity ratio. Quantitative analysis of the green/red fluorescence shows that the NAC and/or AAP decreased green fluorescence, while NAC and/or AAP inhibited H_2_O_2_-mediated mitochondrial permeability. (C) The expression and localization of apoptosis related mitochondrial proteins. Expression of BAX and cytochrome *c* proteins in hADMSCs pretreated with NAC or/and AAP were assessed by western blot. COX-IV and a-tubulin were used as mitochondrial and cytosolic internal controls, respectively. Summary of normalized values of cytochrome *c* and BAX levels in the mitochondrial and cytosolic fractions of hADMSCs were shown at the right. (D) Immunostaining of BAX (left) and cytochrome *c* (right) using a respective FITC-conjugated antibodies (green) and MitoTracker (red) as the indicator of mitochondria in NAC or/and AAP-pretreated hADMSCs. Scale bar = 10 µm. * *p* < 0.05, ** *p* < 0.01, *** *p* < 0.001.

Since caspase-9 and BAX were activated in H_2_O_2_-treated hADMSCs, we further characterized its effect on mitochondrial membrane potential (MMP), the localization of BAX, and the release of cytochrome *c* from mitochondria to cytosol, respectively. In normally polarized mitochondria, JC-1 forms aggregates or monomers depending on the state of MMP. JC-1 forms fluorescent red aggregates in the mitochondria of untreated hADMSCs with polarized or higher MMP, while disperses into fluorescent green monomers in the mitochondria with depolarized or lower MMP. After the pretreatment of NAC or/and AAP, JC-1 gathered in the mitochondrial matrix and produced red fluorescence (Fig. 3A, enlarged view). Further flow cytometric analysis revealed that H_2_O_2_ resulted a dramatic reduction of red fluorescence, indicating a loss of MMP and the damage of mitochondria in hADMSCs (Fig. 3B). The ratio of red and green fluorescence represented the level of depolarization in mitochondria: the NAC/AAP protected hADMSCs against the damage caused by H_2_O_2_ treatment.

Mitochondrial dysfunction can provoke the release of cytochrome *c* from mitochondria into cytosol. Western blot analysis revealed that exposure of hADMSCs to H_2_O_2_ induced a significant increase in cytosolic cytochrome *c*, accompanied by a decrease of cytochrome *c* in the mitochondrial fraction (Fig. 3C). NAC or/and AAP pretreatment also decreased BAX translocation to the mitochondrial membrane. Translocation of BAX to the mitochondrial membrane has been shown to facilitate the release of cytochrome *c*^23^,^24^. In addition, immunostaining also confirmed the cellular distribution of BAX and cytochrome *c* (Fig. 3D). The mitochondria of hADMSCs were primarily identified by MitoTracker, and later immunolabeled with FITC-conjugated BAX (Fig. 3D, left panel) and cytochrome *c* (Fig. 3D, right panel) antibodies, respectively. NAC/AAP suppressed mitochondrial translocation of BAX and reduced cytochrome *c* release from mitochondria to cytosol at the presence of H_2_O_2_. These results confirmed the protective effect of combined treatment of NAC and AAP in preventing mitochondrial dysfunction associated with intrinsic apoptotic pathway.

### NAC/AAP mediated atypical necrosis via AIF translocation in H_2_O_2_-induced cell death

**Figure 4:**
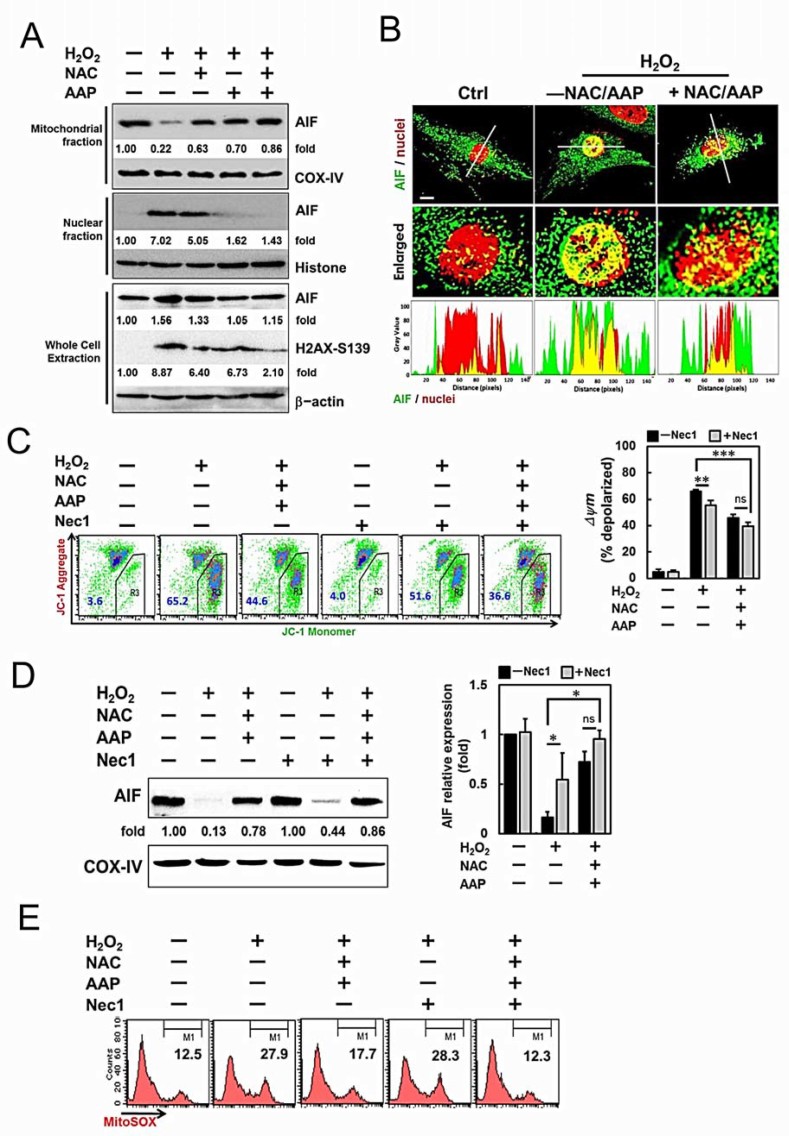
AIF/H2AX pathway was involved in the protective effect of NAC and AAP. (A) Expression of AIF and H2AX proteins in hADMSCs pretreated with NAC or/and AAP were assessed by western blot. COX-IV and Histone were used as mitochondrial and nuclear internal controls, respectively. The changes of each band were expressed as fold-changes as compared to the internal controls. (B) Analysis of fluorescence intensities by confocal microscopy revealed the co-localization of AIF (green fluorescence) in the nuclei (red fluorescence). In the enlarged pictures, the overlapping of the fluorescence (yellow) decreased in NAC/AAP-treated cells. Histograms demonstrate the fluorescence intensity profiles along the lines indicated in the upper row images. (C) Mitochondrial membrane potential changes of hADMSCs treated with NAC/AAP for 4 h in the absence or presence of Nec1 were analyzed by flow cytometry after JC-1 staining. Quantitative analysis of the green fluorescence (JC-1 monomer) showed that the NAC/AAP decreased green fluorescence in the absence or presence of Nec1 (right panel). (D) The expression of AIF protein in the mitochondrial fraction of hADMSCs pretreated with or without NAC/AAP was assessed at the absence or presence of Nec1 by western blot. COX-IV was used as mitochondrial internal control. Summary of normalized values of AIF in the mitochondrial fractions of hADMSCs.is shown at the right panel. (E) Mitochondrial ROS is shown in the representative histogram of unfixed cells analyzed by flow cytometry after MitoSOX staining. The values indicate the percentage of cells in the marked (M1) regions. Scale bar = 10 µm. * *p* < 0.05, ** *p* < 0.01, *** *p* < 0.001. ns, not significant.

When hADMSCs were pretreated with the NAC or/and AAP before H_2_O_2_ challenge, the activation of executioner, i.e., caspases, was significantly suppressed, and consequently decreasing the cleavage of PARP1 (Fig. 2B). Instead of typical necrotic cell morphology, some dying cells that swelled and showed a “bubbled-like” nuclear morphology resembling apoptosis were also observed (Fig. 2A, upper panel). In addition, the flow cytometric analysis also revealed an Annexin V^+^/PI^+^ cell population (Fig. 2A, lower panel). Programmed necrotic cell death or necroptosis is characterized by plasma membrane rupture, permeable to PI, and depolarization of mitochondrial membrane^25^. Approximately 10-20% of these Annexin V^+^/PI^+^ cells induced by H_2_O_2_ could be suppressed by NAC or/and AAP pretreatment (Fig. 2A, lower panel). Loss of MMP is associated with the opening of the mitochondrial transition pore, release of inner mitochondrial components (e.g., apoptogenic proteins), and subsequently apoptosis^26^. Recently it was reported that caspase-independent necroptosis mediated by AIF could promote chromatinolysis, which was associated with the phosphorylation on Ser139 of histone protein H2AX (γH2AX)^27^,^28^. We investigated whether the translocation of AIF and phosphorylation and H2AX occurred in the H_2_O_2_-induced necroptosis of hADMSCs. Immunoblotting revealed that AIF mostly resided in the mitochondria of normal hADMSCs (Fig. 4A). AIF translocation occurred and subsequently appeared in the nuclear fraction of hADMSCs treated with H_2_O_2_ for 4 hr. NAC or/and AAP pretreatment significantly decreased AIF levels in the nuclear fraction upon H_2_O_2_ treatment. As shown in (Fig. 4B), H_2_O_2_ resulted in a significant increase of AIF in the nucleus, while NAC/AAP pretreatment reduced H_2_O_2_-induced AIF translocation to the nucleus. The Ser139-H2AX in the whole cell lysate of hADMSCs could be induced by H_2_O_2_, and was decreased upon NAC/AAP pretreatment (Fig. 4A).

To investigate whether AIF pathway was involved in the H_2_O_2_-induced necroptosis, we introduced Necrostatin 1 (Nec1), an necroptosis inhibitor that blocks RIPK1 activation^25^, before the H_2_O_2_ treatment. The results showed that Nec1 alone had no effect on the mitochondrial AIF level. However, Nec1 treatment decreased the mitochondrial AIF level to 0.44-fold in hADMSCs suffering from H_2_O_2_ challenge (normalized against COX-IV), (Fig. 4D). Addition of NAC/AAP further enhanced the effect of Nec1 on reducing mitochondrial AIF release after H_2_O_2_ treatment (0.86 fold of untreated control). Retaining of AIF, as well as the mitochondrial function was further confirmed by JC-1 staining: NAC/AAP plus Nec1 pretreatment markedly increased the cell population with higher MMP after H_2_O_2_ challenge (Fig. 4C).

The mitochondrial ROS generation was also analyzed with MitoSOX to address the role of NAC/AAP pretreatment in H_2_O_2_-induced necrotopsis (Fig. 4E). Cells under H_2_O_2_ treatment showed higher red fluorescence as compared with that in the normal control group (27.9% *vs.* 12.5%). The fluorescence intensity was lower in the NAC/AAP pretreated cells (17.7%), while Nec1 treatment did not reduce the mitochondrial ROS production (28.3%). More remarkably, the Nec1 and NAC/AAP co-treatment totally suppressed the mitochondrial ROS production (12.3%).

### NAC/AAP inhibited the formation of necrosome in H_2_O_2_-induced cell death.

**Figure 5:**
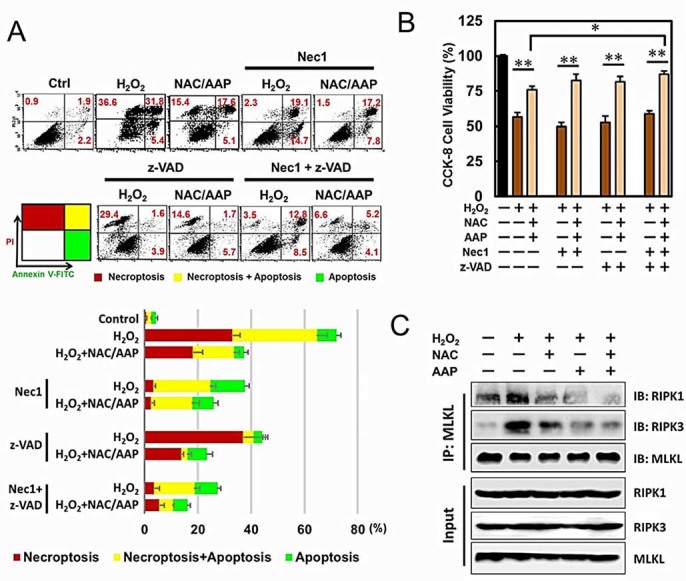
NAC/AAP attenuated necroptosis at the presence of caspase and RIPK1 inhibitors. (A) hADMSCs were treated with NAC/AAP at the presence or absence of Nec1 (100 mM) and z-VAD (50 mM) for 120 min following by H_2_O_2_ treatment. Cells stained with Annexin V-FITC and PI were analyzed by flow cytometry. The values indicate the percentage of cells in each region were summarized at the lower panel. (B) The survival of hADMSCs with NAC/AAP pretreatment at the presence or absence of Nec1 and z-VAD following by H_2_O_2_ treatment. (C) hADMSCs were treated with NAC or/and AAP followed by H_2_O_2_ stimulation. MLKL was immuneprecipitated and immunoblotted. * *p* < 0.05, ** *p* < 0.01.

To further delineate the protective mechanism of NAC/AAP in suppressing H_2_O_2_-induced necroptosis and apoptosis, we tested the effect of the RIPK1 inhibitor (Nec1) and pan-caspase inhibitor (z-VAD) on these H_2_O_2_-treated hADMSCs. Previous studies have shown that Annexin V and PI staining can be used to assess different cell deaths: Annexin V^+^/PI^-^ cells were regarded as early apoptotic cells, and double positive cells were regarded as late apoptotic or necroptotic cells^29^. Apoptosis and necroptosis are mediated by distinct but overlapping pathways involving cell surface death receptors and cellular components^30^. Different types of cell death were classified in cytometric analysis according to Annexin V and PI staining pattern (Fig. 5A): the Annexin V^+^/PI^-^ cells were early apoptotic cells (green region), the Annexin V^+^/PI^-^ cells were necroptoptic cells (Nec1 sensitive cells in the red region), and double positive cells represented cells that underwent both apoptosis and necroptosis (yellow region). The results demonstrated that NAC/AAP partially inhibited H_2_O_2_-induced necroptosis in hADMSCs (Fig. 5A, 35.4% reduction). Addition of Nec1 almost completely blocked H_2_O_2_-induced necroptosis in hADMSCs with or without NAC/AAP pretreatment (21.4% and 18.7% reduction, respectively). However, Nec1 did not inhibit early apoptosis (5.4% *vs.* 14.7%). NAC/AAP inhibited apoptosis in H_2_O_2_-treated hADMSCs (14.5% reduction), however it did not augment the effect of z-VAD which would inhibit caspases activation during apoptosis (3.9% *vs.* 5.7%). When both Nec1 and z-VAD were incubated with hADMSCs prior to H_2_O_2_, all types of cell death were mostly suppressed. Interestingly Nec1 plus z-VAD, together with NAC/AAP, almost completely abolished the H_2_O_2_-induced necroptosis and apoptosis cell death (Fig. 5A). Co-treatment of NAC/AAP significantly increased cell viability from 56.3% (H_2_O_2_-treated group) to 75.9% (NAC/AAP-treated group), while in combined with Nec1 and z-VAD increased cell survival from 75.9% (NAC/AAP-treated group) to 86.9% (Nec1+z-VAD+NAC/AAP group) (Fig. 5B). Previous studies showed that the interference of RIPK1, RIPK3, and MLKL could block the assembly of the necrosome, and hence the necroptosis that led to cellular swelling and plasma membrane rupture^25^. To further elucidate the mechanism of necroptosis in H_2_O_2_-induced cell death, we analyzed the assembly of necrosome under various treatments by immuno-precipitation. The results showed that H_2_O_2_ treatment significantly increased the formation of necrosome as revealed by the increase of RIPK1 and RIPK3 in MLKL-immunoprecipitate (Fig. 5C). Taken together, these findings suggested that NAC/AAP inhibited the formation of necrosome in H_2_O_2_-induced necroptosis in hADMSCs.

### NAC/AAP attenuated mitochondrial dysfunction and restored mitochondrial morphology during H_2_O_2_ treatment

**Figure 6:**
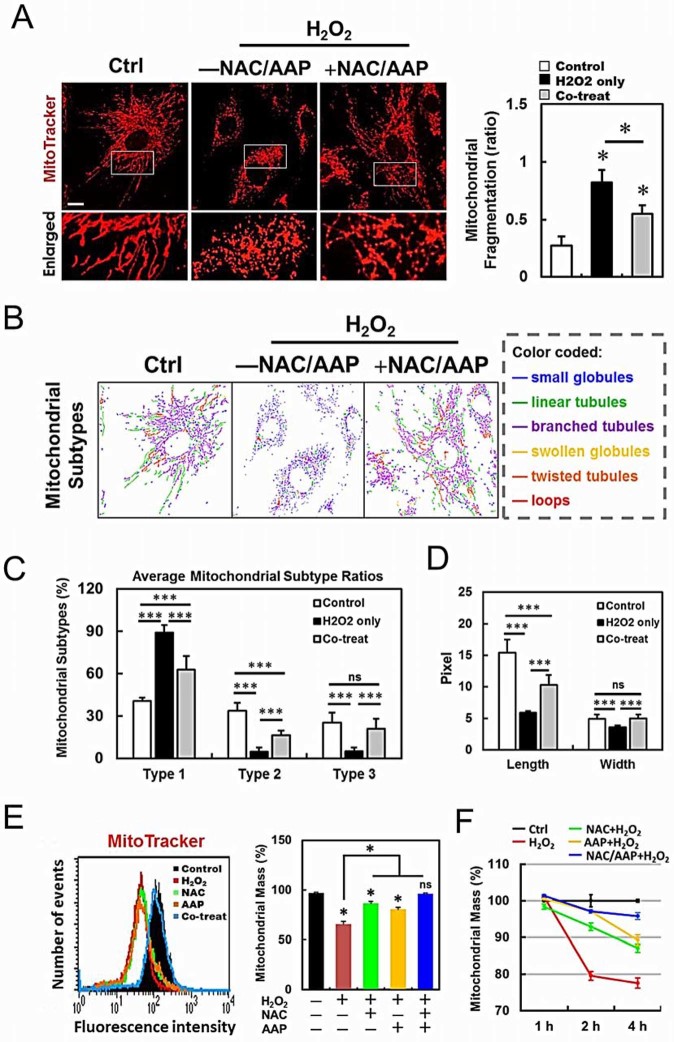
NAC/AAP protected mitochondria against oxidative stress. (A) hADMSCs were stained with MitoTracker Red and observed by confocol microscopy to analyze the mitochondrial network structure. The acquired images were filtered, and thresholded to identify mitochondrial fragments using ImageJ software. The particle number was normalized to the total mitochondrial area to calculate the MFC (mitochondria fragmentation counting). The ratio changes are shown at the right panel. (B) MicroP software classifies mitochondria in each cell into three categories according to the features of mitochondrial morphology. (C and D) The proportions of different categories of mitochondria obtained from various treatments are shown in (C), and the changes in length and width of the mitochondria are shown in (D). The effects of NAC or/and AAP protection on mitochondrial mass are shown in E: hADMSCs of various treatments were stained with MitoTracker Red, and their mitochondrial mass were measured by flow cytometry at different time points (F). The data were expressed as a percentage to the non-treated control, which was set at 100%. Scale bar = 10 µm. * *p* < 0.05, ** *p* < 0.01, *** *p* < 0.001. ns, not significant.

To assess whether NAC/AAP treatment was sufficient to protect mitochondria from H_2_O_2_-induced damage, we analyzed the mitochondrial network using confocal microscopy. After visualizing the mitochondria with fluorescent MitoTracker Red, we found that mitochondrial fragmentation was markedly increased after H_2_O_2_ treatment (Fig. 6A). Notably, pretreatment of NAC/AAP efficiently inhibited the increase of mitochondrial fragmentation caused by H_2_O_2_. We further classified the mitochondrial morphological changes with MicroP software^31^. The mitochondria were classified into three types according to the characteristics of its morphology (Fig. 6B): small globular (Type 1), linear tubular (Type 2), and branched/twisted tubular, swollen globular or loops (Type 3). Our data showed that NAC/AAP-pretreated hADMSCs displayed a significantly higher percentage of types 2 and 3 mitochondria than the non-pretreated cells (Fig. 6C). In addition, after the H_2_O_2_ insult, the average length and width of the mitochondria in NAC/AAP-pretreated cells significantly outscored those without pretreatment (Fig. 6D). To investigate whether the protection of NAC/AAP on mitochondria was related to the duration of H_2_O_2_ exposure, we treated cells with H_2_O_2_ for different time periods and then stained mitochondria with MitoTracker. We observed that cells treated with both NAC/AAP had 96% protection in maintaining the mitochondrial mass at 2- and 4 hr, respectively, after H_2_O_2_ exposure (Fig. 6E & 6F). These results suggested that NAC/AAP reduced mitochondrial fission and helped maintaining their mass.

### NAC/AAP reduced mitochondrial fission via regulating Drp1 translocation during H_2_O_2_ treatment

**Figure 7:**
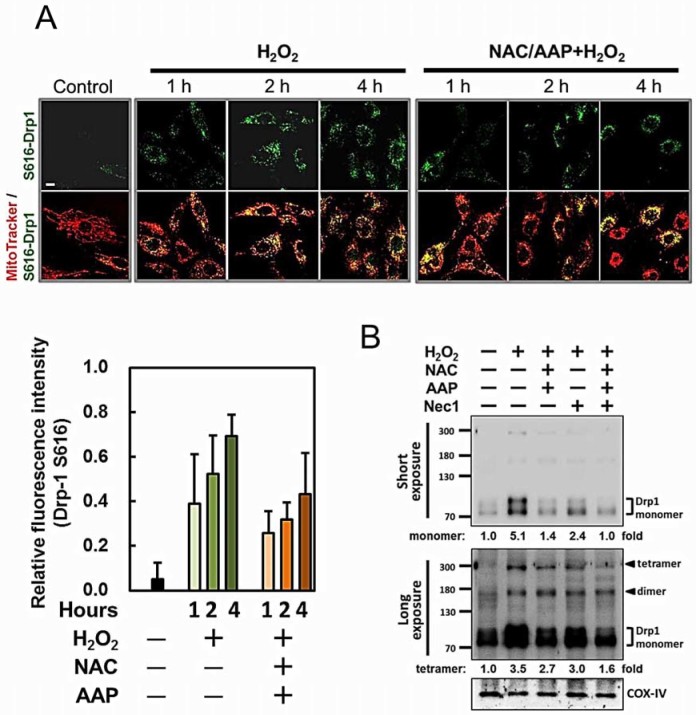
NAC/AAP decreased the activation of Drp1 in H_2_O_2_-treated hADMSCs. (A) The co-localization of Drp1 Ser616 (green) in the mitochondria (MitoTracker, red) was revealed by confocal microscopy. The merged images clearly show the recruitment of Drp1 Ser616 to mitochondria in response to H_2_O_2_ treatment. NAC/AAP pretreatment partially prevented mitochondrial translocation of Drp1 S616 induced by H_2_O_2_ in a time-dependent manner. The fluorescence data were quantified by ImageJ software. Quantification data were obtained from at least five independent experiments (lower panel). (B) Western blot analysis was employed to study the translocation of Drp1 to mitochondria in response to H_2_O_2 _treatment. The protein levels of Drp1 were decreased by Nec1 pre-incubation. Long exposure revealed ~160 and ~300 kDa bands in the mitochondrial fraction. Scale bar = 10 µm.

Recruitment and/or retention of Drp1 in the mitochondria have been implicated in mitochondrial fission and mitopotsis^32^,^33^. We further explored the possible mechanism of how NAC/AAP reduced mitochondrial fission by monitoring Drp1 phosphorylation. Using an antibody specifically recognizing phosphorylated Drp1 at Ser616 (Drp1 S616), we showed that the level of Drp1 S616 was decreased, as well as its translocation to mitochondria by NAC/AAP pretreatment (Fig. 7A, upper panel). It was found that, under H_2_O_2_ treatment, Drp1 S616 showed punctate distribution within the mitochondrial networks. NAC/AAP pretreatment reduced 1/3 of Drp1 S616 translocation as revealed by the decreased of Drp1 S616 staining over the mitochondria (i.e., FITC fluorescence/MitoTracker, Fig. 7A, lower panel).

The polymerization of Drp1 could cause mitochondrial fragmentation, an early and obligatory step for necroptosis execution. As shown in Figure 7B, western blot analysis revealed that exposure of hADMSCs to H_2_O_2_ induced an increase in mitochondrial Drp1 levels, accompanied by a parallel increase of high molecular weight Drp1 reactive bands (i.e., the Drp1 dimer and tetramer) in the longer exposure image. These findings indicated that NAC/AAP suppressed Drp1-mediated mitochondrial fission.

## Discussion

The rationale for using combination treatment with NAC and vitamin C to prevent cell death and injury of tissues due to oxidative stress is largely based on in vitro observations. This may be particularly relevant in preventing cell death and injury due to H_2_O_2_ exposure. However, vitamin C is in fact an important ingredient of the osteogenic differentiation medium, and has been reported to increase cell proliferation and differentiation of BMMSCs into osteocytes and adipocytes^34^. Langenbach and Handschel reviewed and concluded that vitamin C could lead to the increased secretion of collagen type I (Col1), which in turn upregulated Col1/α2β1 integrin-mediated intracellular signaling. Activation of the Col1/α2β1 signaling pathway facilitates the osteogenic process that is initiated by dexamethasone^35^. Cao *et al.* also demonstrated that vitamin C enhanced the cardiac differentiation of induced pluripotent stem cells via promoting the proliferation of cardiac progenitor cells^34^,^36^. Previous reports have also shown the addition of other antioxidants, such as epigallocatechin-3-gallate (EGCG), curcumin, melatonin and β-estradiol, can reduce cellular oxidative stress and promote the proliferation of MSCs^37^,^38^,^39^.

We previously showed that addition of NAC/AAP could modulate the cell cycle progression of hADMSCs by downregulating CDK inhibitors: at the presence of NAC/AAP, cells proliferated more rapidly, yet retained their stemness and their differentiation ability^21^. Interestingly, the NAC/AAP-induced changes in hADMSCs were quite similar to those cultivated under hypoxia (1%–5% pO_2_)^22^, suggesting that NAC/AAP might activate similar biochemical pathways that lead to cell survival. Taken together, these observations led to the hypothesis that combination treatment of vitamin C with ROS inhibitor (i.e., NAC) enhances free radical scavenging, and might protect them from the adverse effects of excessive oxidative stress.

In this study, we first identified the proper combination of NAC and AAP (3 mM and 0.2mM, respectively) that exerted maximum protection on hADMSCs that were under H_2_O_2_-induced oxidative stress (Supplementary Table S1 & Fig. 1E). The NAC/AAP-treated cells have less mitochondrial ROS (Figs. 4E), which we believe is beneficial to mitochondrial function (Figs. 3A & 3B) and integrity (Fig. 6). Mitochondrial morphology is tightly controlled by the balance between mitochondrial fission and fusion^14^. Our data demonstrated that NAC/AAP reduced mitochondrial fragmentation as characterized by decreased fission, increased fusion, or both. In support of this point, we demonstrated that the types of mitochondria in the NAC/AAP-treated cells were nearly the same as we observed in the control group (Fig. 6B). At the molecular level, H_2_O_2_ treatment also resulted in Drp1 phosphorylation and translocation to mitochondria, while NAC/AAP pretreatment significantly reduced both changes (Fig. 7).

Stabilization of mitochondrial function by NAC/AAP pretreatment was revealed by the JC-1 staining (Fig.3A & 3B). It is known that mitochondria MMP plays diverse roles in cell physiology and pathology including regulations of necroptosis and mitoptosis^40^,^41^. The inhibition of mitochondrial MMP depolarization in hADMSCs may prevent the breakdown of mitochondrial integrity thus restrict the activation of internal mitochondrial-dependent apoptosis and the release of other cell death factors (e.g., cytochrome c, AIF, etc.).

Mitoptosis is defined as a form of mitochondrial programmed cell death (PCD). It could be associated with both necrosis and apoptosis, although regressing mitochondria are also found in autophagic vacuoles^42^. In our study, NAC/AAP inactivated RIPK1 and RIPK3 that led to the reduction of H_2_O_2_-induced necroptosis (Fig. 5). In addition, NAC/AAP reversed H_2_O_2_-induced mitoptosis and necroptosis in hADMSCs, and Nec1 pre-incubation decreased the expression of Drp1 protein induced by H_2_O_2_ treatment. The data suggest that NAC/AAP may directly or indirectly affect necroptosis through mitochondrial fission in hADMSCs. Moreover, our data demonstrate for the first time both mitoptosis and necroptosis pathways contribute to the protection effect of NAC/AAP on hADMSCs under H_2_O_2_.

Mitochondrion is well known for its function in PCD and is also involved in the down-stream regulation of PARP1^43^. PARP1 has been implicated in two modes of cell death induced by DNA damage, namely apoptosis and necroptosis^44^. Typically, PARP1 can be activated by DNA breaks, cellular stresses^45^, and the posttranslational modifications such as phosphorylation, acetylation or PARylation^46^. The PARylation of proteins is thought to exhaust cells’ ATP and NAD that subsequently leads to necroptosis, and PARPs also induce AIF release from mitochondria to nuclei^47^. A role for AIF in the permeabilization of mitochondrial membranes and the translocation of cytotoxic proteins has been proposed in necroptosis^28^. Besides the activation of PARP1 and BCL2 family, the apoptogenic form of AIF is associated with Ser139-H2AX phosphorylation^27^,^28^,^48^. Our result demonstrated that NAC/AAP pretreatment reduced the phosphorylation of H2AX (Fig. 4A). Notably, a single pretreatment of hADMSCs with AAP was sufficient to preclude AIF nuclear translocation (Fig. 4A). Two signal transducers of DNA damage, namely PARP1 and H2AX, are both involved in the regulation of necroptosis: they are both activated in response to DNA damage and are implicated in PCD^49^,^50^.

**Figure 8:**
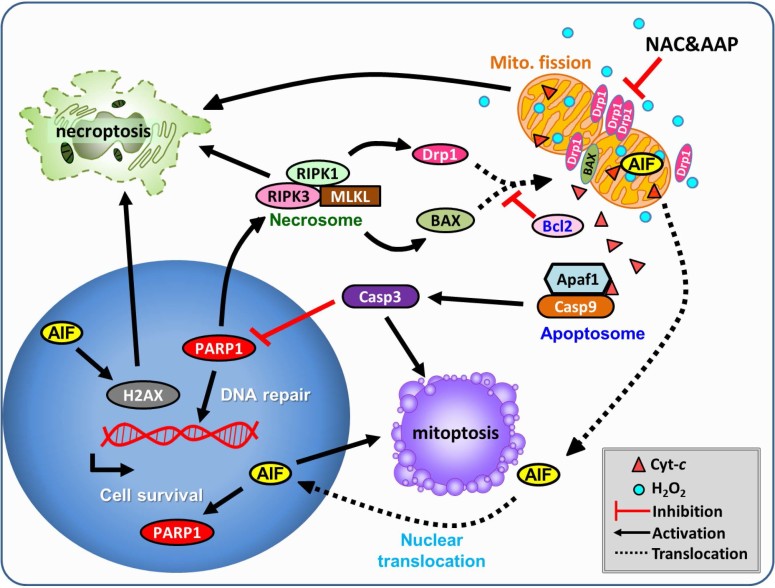
Scheme illustration of how NAC and AAP protect mitochondria against oxidative damage in hADMSC, and subsequently rescue the cell from necroptosis and apoptosis. Both NAC and AAP protect cells from oxidative stress-induced cell death via inhibiting mitoptosis and necroptosis in hADMSCs. NAC/AAP suppress necroptosis via decreasing the translocation of AIF, and thus stopping the formation of necrosome. Stabilization of mitochondrial integrity prevents the secretion of apoptotic factors that are resided in the mitochondria.

With regard to the direct inhibitory effect on cells, our data showed that NAC/AAP could inhibit the translocation of BAX (Figs. 3C & 3D) and Drp1 (Fig. 8) to the mitochondria. It has been shown that other key molecular mediators including cytochrome *c*, AIF, Drp1, and RIPKs also promote necroptosis by activating multiple signal pathways (Fig. 8). RIPK1 and RIPK3 can act as lethal effectors in necroptosis: mixed lineage kinase domain-likeprotein (MLKL), phosphoglycerate mutase family member 5 (PGAM5), and the fission mediator Drp1^51^,^52^. Indeed, we confirmed that NAC/AAP elicited the activation of multiple mitoptotic signal cascades, such as Drp1/BAX/caspase-dependent and Drp1/BAX/caspase-independent pathways. Our data showed that NAC/AAP decreases necroptosis without the induction of ROS generation (Fig. 4E), indicating that combined therapy may directly or indirectly regulate multiple signal transduction pathways.

## Conclusion

The synergistic protective mechanism of NAC/AAP to suppress H_2_O_2_-induced necroptosis, mitoptosis, and apoptosis in hADMSCs is illustrated in (Fig. 8). Our results demonstrate that NAC/AAP diminish BAX and Drp1 translocation from cytoplasm to mitochondria, and jointly contribute to mitochondrial integrity. Maintenance of mitochondrial function is accompanied with decrease of ROS production and thereby protects hADMSCs from mitoptosis. As mitochondria are protected in hADMSCs after treating with NAC/AAP, down-regulation of AIF, H2AX, and PARP1 occur, and may subsequently activate genes that are involved in the synthesis and repair of DNA. However, enhanced proliferation through inhibition of necroptotic can also be another possible mechanism to explain the synergistic effects of NAC/AAP.

## Methods

### Isolation and maintenance of hADMSCs

This study was approved by the Buddhist Tzu Chi General Hospital Institutional Review Board (IRB102-130): hADMSCs were isolated from the human adipose tissue left over using our previously published method^21^. The hADMSCs were cultured in MSC maintenance medium containing Iscove’s modified Dulbecco’s medium (IMDM), 10% fetal bovine serum (FBS, MSC-Qualified), 0.1 M sodium bicarbonate, 2 mM L‐glutamine (all from GIBCO-Invitrogen Co., CA, USA) and 10ng/mL FGF-2 (R&D Systems, MN, USA) at 37°C in a humidified incubator containing 5% CO_2_ and 95% air. All experiments were performed on hADMSCs from passage 3 to 6.

### *Ethics statement*

The institutional review board at Buddhist Tzu Chi General Hospital approved all study procedures. The study was performed in accordance with approved guidelines. Written informed consent was obtained from each patient and/or guardians. The study was carried out in compliance with the Helsinki Declaration.

### *Cell treatment*

The cells cultured in complete medium were used as the normal control. For NAC and AAP (all purchased from Sigma Co., MO, USA) co-treatment experiment, the hADMSCs were pretreated with various concentrations of NAC or AAP for 20 h and followed by incubation in medium containing 0.5mM H_2_O_2_ (Sigma Co., MO, USA) for 4h (Figs. 1A & 1B). The concentrations that exert maximum protection were combined and further tested for their protection effect. For inhibitor studies, the cells were incubated with indicated amount of NAC or/and AAP for 20 h, and subsequently 2 h with medium containing a receptor-interacting protein kinase 1 (RIPK1) inhibitor, necrostatin-1 (Nec-1, 0.1mM, ab141053; Abcam, MA, USA) or/and a pan-caspase inhibitor z-VAD-FMK (0.01 mM, ab120382; Abcam, MA, USA) prior to the final 0.5 mM H_2_O_2_ challenge.

### Cell proliferation assay

Cell proliferation was measured for cells growth in 96-well plates by using Alamar Blue (AbD Serotec, Oxford, UK), an oxidation-reduction indicator. The absorbance of Alamar Blue is correlated with cell number. Briefly, 10% of Alamar Blue was added to the seeded cells for 4 h. The absorbance of this reaction was read at 570 and 600nm in a multi-well plate ELISA reader (Bio-Tek Instruments, VT, USA). Cell proliferation (% of untreated control) was calculated with the following equation:

Proliferation (%) = [(117216×OD570 (sample)-80586×OD600 (sample)] / [(117216×OD570 (control)-80586×OD600 (control)]×100%; 117216 and 80586 are the molar extinction coefficients of reduced- and oxidized- Alamar Blue, respectively.

### Calculation of combination index and dose reduction index

The Chou-Talalay method has been used in drug combination studies and it can provide an estimation of drug synergistic effect by calculating the combination index (CI): CI < 1 indicates synergistic effect, CI = 1 indicates additive effect, and CI > 1 indicates antagonism. The CI is calculated as: CI = (*D*)_1_/(*Dx*)_1_+(*D*)_2_/(*Dx*)_2_, where (*Dx*)_1 _and (*Dx*)_2_ indicate the mono protection doses of NAC and AAP, respectively; while (*D*)_1_ and (*D*)_2_ are the doses of NAC and AAP that can cause the similar protection effect in combination. The dose reduction index (DRI) is defined by the level of dose reduction that is possible in a combination for a given level of effect as compared with the concentration of individual drug alone. The equation of the DRI can be shown as: (DRI)_1_ = (*Dx*)_1_/(*D*)_1_ and (DRI)_2_ = (*Dx*)_2_/(*D*)_2_^53^.

### Annexin V-FITC/PI double staining

Apoptotic cell death was measured by Alexa Fluor Annexin V/Dead Cell Apoptosis kit (Molecular Probes Inc., Eugene, OR, USA) according to the manufacturer’s protocol. Cells were harvested after various treatments, washed twice with cold binding buffer, resuspended in binding buffer and stained with 5 mL of Annexin V-FITC and propidium iodide (PI) in dark for 15 min at room temperature. After incubation, 1 mL binding buffer was added, and cells were analyzed by flow cytometry (FACSCalibur, BD Bioscience, CA, USA).

### Cell viability assay

Cell viability was analyzed using CCK-8 (Cell Counting Kit-8, Enzo Life Sciences Inc., NY, USA) that detected the metabolic activity of viable cells. Cells were plated at a density of 2×10^3^ cells/well in 96-well plates with the complete medium. At the end of various treatments, 10 μL of the CCK-8 reagent was added to each well and incubated at 37°C for 4 h. Absorbance was recorded by an ELISA microplate reader at 450nm.

### Cellular production of ROS

Intracellular ROS (composed mainly of hydrogen peroxide and superoxide anion) was measured using Total ROS/Superoxide Detection Kit (Enzo Life Sciences Inc., NY, USA). After incubation with H_2_O_2_ for indicated times, cells were stained with the detection reagent at 37°C for 30 min. ROS production of hADMSC cells was analyzed by fluorescence microscopy (Carl Zeiss Axiovert M200, Jena, Germany) and flow cytometry, respectively.

### Mitochondrial membrane potential, ROS and mass measurement

Cells were harvested after various treatments, washed twice with PBS, resuspended in culture medium and stained with JC-1 reagent (10mg/mL), MitoSOX reagent (5mM) and MitoTracker Green FM or MitoTracker Red CMXRos (50nM) (all from Molecular Probes Inc., Eugene, OR, USA) at 37°C for 30 min. After incubation, 1mL PBS was added, and cells were analyzed by flow cytometry.

### Subcellular fractionation, protein extraction, immunoprecipitation, and immunoblotting

Cytosolic, nuclear, and mitochondrial fractions of the cells were isolated with NE-PER Nuclear & Cytoplasmic Extraction Reagents and Mitochondria Isolation Kit (both from Thermo Scientific, MA, USA), respectively, according to the manufacturer’s recommendation. Cells from various treatments were lysed with RIPA buffer (Millipore Co., MA, USA) and sonicated on ice for 5 min. After centrifugation at 13,000 x *g* for 15 min at 4°C, the supernatant was transferred to a fresh tube and the resulting protein concentration was determined by Bradford protein assay (Protech Inc., Taipei, Taiwan) with bovine serum albumin (BSA) as the standard. For immunoprecipitation study, total protein (0.2mg) was incubated with anti-mixed lineage kinase domain-like (MLKL, Millipore Co., MA, USA) antibody at 4°C overnight. Then, 100µL of pre-cleared protein G-Beads (Millipore Co., MA, USA) was added and incubated at 4°C for 2 h. The resulting immunoprecipitate was boiled with SDS reducing sample buffer and subjected to SDS-PAGE. After electrophoresis, protein was blotted onto a PVDF membrane (Millipore Co., MA, USA) and blocked with skim milk at room temperature for 1 h. Each membrane was incubated with appropriate primary antibodies at 4°C overnight. The blots were incubated with HRP-conjugated secondary antibodies for 1 h, washed 3 times with PBST (PBS containing 0.1%Tween-20), visualized by Immobilon Western Chemiluminescent HRP Substrate (Millipore Co., MA, USA), and images recorded with the Keta Luminescent image analyzer (Wealtec Bioscience Co., Taipei, Taiwan).

### Cell morphology examination

Cell morphology was examined by Hoechst 33258 and PI double staining for live-cell imaging. After various treatments, cells were stained with Hoechst 33258 and PI solution at room temperature for 10 min. Cells were washed twice with PBS and observed with inverted fluorescence microscope (Carl Zeiss Axiovert M200).

### Immunofluorescence labeling

Cells were washed with PBS and incubated with 50nM MitoTracker Red CMXRos at 37°C for 30 min. To determine the subcellular localization of BAX, cytochrome *c* and phosphorylated-Drp-1 at Ser616 (Drp-1 S616), cells were washed twice with PBS, fixed with 4% paraformaldehyde in PBS and subsequently permeabilized with 0.2% Triton X-100 on ice for 5 min. After washing with PBS twice, cells were incubated in blocking solution (PBS containing 20% goat serum) at room temperature for 30 min and incubated with primary antibodies at 4°C overnight. After washing with PBS, the cells were then stained with FITC-conjugated secondary antibodies (1:250, Code 711-545-152, Jackson ImmunoResearch Lab, Inc., PA, USA), counterstained with Hoechst 33258 for 5 min, and visualized by confocal laser scanning microscopy (LSM510 Meta, Carl Zeiss, Jena, Germany).

### Antibodies and Inhibitors

Anti-PARP1 (100573), anti-BAX (109683), anti-BCL2 (100064), anti-cytochrome c (108585), anti-COX-IV (101499), anti-a-tubulin (112141), anti-RIPK1 (111074), anti-RIPK3 (107574), and anti-histone H3 (122148) were purchased from GeneTex (ICON-Genetex Inc., Taipei, Taiwan); Anti-caspase-9 (ab115161) and anti-caspase-3 (ab90437) were purchased from Abcam. Anti-MLKL (MACB604) was purchased from Millipore. Anti-AIF (#4642), anti-Drp1 (#5391), anti-Drp1 Ser616 (#3455) and anti-phospho-H2AX (Ser139) (#9718) were purchased from Cell Signaling Technology (MA, USA). Anti-b-actin (A5411) was purchased from Sigma.

### Statistical analysis

The intensity of bands in Western blots or fluorescent images were quantified by using AlphaDigiDoc (Cell Biosciences, ON, Canada), ImageJ (NIH), or MicroP software^31^. The intensity values were normalized against the intensity of the loading control for the same sample. The values after normalizing to loading control in the control groups were set as 1.0. All values were expressed as mean ± standard error of the mean (SEM) and were analyzed using a Student’s *t*-test with two-tailed distribution between groups as indicated in the graphs. All calculations were performed by Microsoft Excel 2010.

## Author Contributions

C.J.L. performed experiments, data analysis and wrote the manuscript. L.Y.S. provided conceptual input. C.Y.P. designed the experiments, supervised the research and revised manuscripts. All authors reviewed the final version of the manuscript.

## Supplementary Material

Supplementary Information Supplementary Information
