# Supplementary material for: Synergistic Protection of N-Acetylcysteine and Ascorbic Acid 2-Phosphate on Human Mesenchymal Stem cells Against Mitoptosis, Necroptosis and Apoptosis
Source: Sci Rep. 2015 Apr 24;5:9819. doi: 10.1038/srep09819 (PMC4408980; doi:10.1038/srep09819)
Supplement: Supplementary Information [file srep09819-s1.pdf]

Supplementary information

Synergistic Protection of N-Acetylcysteine and Ascorbic Acid 2-Phosphate on Human Mesenchymal Stem cells Against Mitoptosis,Necroptosisand Apoptosis

Chia-Jung Li[1], Li-Yi Sun[2], Cheng-Yoong Pang[1,2*]

[1]Institute of Medical Sciences, Tzu Chi University, Hualien, Taiwan

[2]Department of Medical Research, Buddhist Tzu Chi General Hospital, Hualien, Taiwan

Supplementary Fig. S1

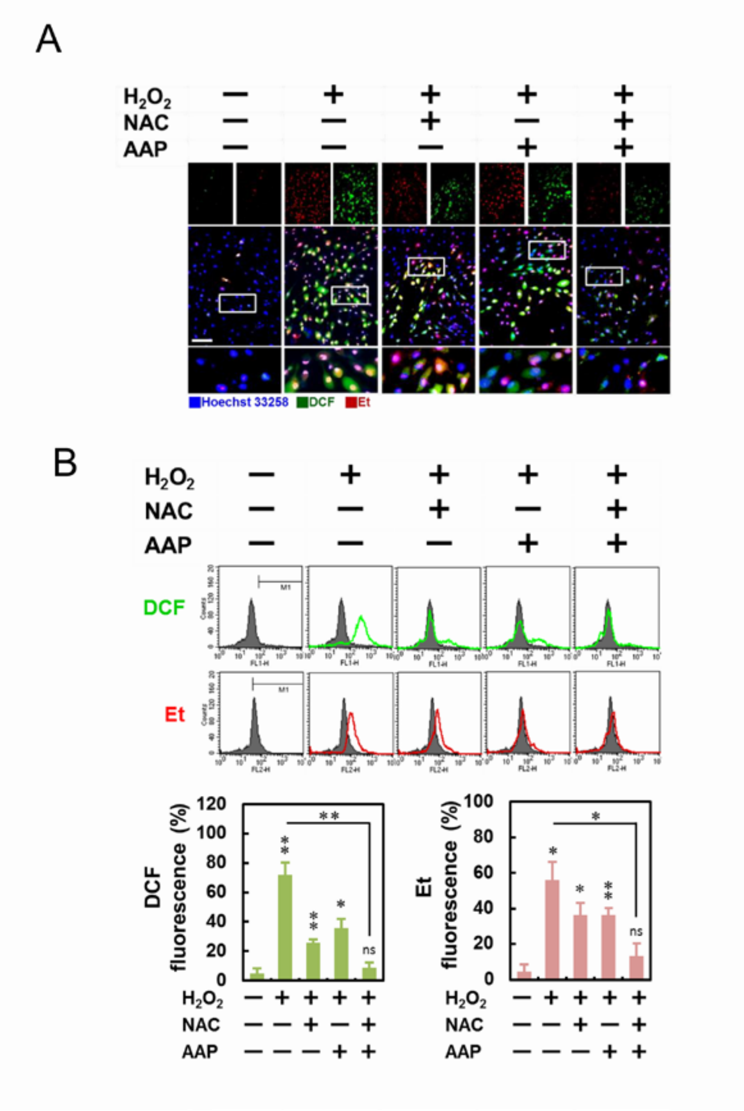

Supplementary Fig. S1 NAC/AAP suppressed the generation of intracellular ROS in H_2O_2-treated hADMSCs

(A) hADMSCs were pre-treated with NAC and/or AAP followed by H_2O_2 challenge. After various treatments, the cells were stained with DCFDA (green) or DHE (red), respectively, and counterstained with Hoechst 33258 (blue); the level of ROS was detected by fluorescence microscope. Scale bar = 100 μm. (B) Flow cytometric analysis of the ROS in various groups of NAC- and/or AAP-treated hADMSCs. Quantitative data were depicted and analyzed from the FL1-H and FL2-H of each histogram. The changes of fluorescence depicted from DCF (oxidized form of

DCFDA) and Et (oxidized form of DHE), respectively, were statistically significant between the treated-groups and the non-treated control. * $p < 0.05$, ** $p < 0.01$, ns, not significant.

Supplementary Table S1. Combination index (CI) and Dose reduction index (DRI) for drug combination by NAC and AAP.

Percentage of protection		NAC Concentration (mM)			AAP Concentration (mM)		
		Alone	Mix		Alone	Mix	
**Drug combinations IC_{25}**	**CI**			**DRI**			**DRI**
**Group 1**	**0.893**	**7**	**1**	**7.0**	**0.8**	**0.6**	**1.3**
**Group 2**	**0.911**	**7**	**2**	**3.5**	**0.8**	**0.5**	**1.6**
**Group 3**	**0.679**	**7**	**3**	**2.3**	**0.8**	**0.2**	**4.0**
**Group 4**	**0.804**	**7**	**3**	**2.3**	**0.8**	**0.3**	**2.7**
**Group 5**	**0.786**	**7**	**2**	**3.5**	**0.8**	**0.4**	**2.0**
**Group 6**	**0.929**	**7**	**3**	**2.3**	**0.8**	**0.4**	**2.0**
**Group 7**	**1.196**	**7**	**4**	**1.8**	**0.8**	**0.5**	**1.6**

CI and DRI represent the fold of dose reduction that is allowed in combination for a given degree of effects as compared with the dose of each drug alone.
